# Discriminant haplotypes of avirulence genes of *Phytophthora sojae* lead to a molecular assay to predict phenotypes

**DOI:** 10.1111/mpp.12898

**Published:** 2020-01-07

**Authors:** Chloé Dussault‐Benoit, Geneviève Arsenault‐Labrecque, Humira Sonah, François Belzile, Richard R. Bélanger

**Affiliations:** ^1^ Department of Phytology Université Laval Québec QC Canada; ^2^ National Agri‐Food Biotechnology Institute (NABI) Mohali India

**Keywords:** *Avr* genes, effectors, multiplex polymerase chain reaction, plant pathogen, phytophthora root rot (PRR), *Rps* genes, soybean diseases

## Abstract

The soybean–*Phytophthora sojae* interaction operates on a gene‐for‐gene relationship, where the product of a resistance gene (*Rps*) in the host recognizes that of an avirulence gene (*Avr*) in the pathogen to generate an incompatible reaction. To exploit this form of resistance, one must match with precision the appropriate *Rps* gene with the corresponding *Avr* gene. Currently, this association is evaluated by phenotyping assays that are labour‐intensive and often imprecise. To circumvent this limitation, we sought to develop a molecular assay that would reveal the avirulence allele of the seven main *Avr* genes (*Avr1a*, *Avr1b*, *Avr1c*, *Avr1d*, *Avr1k*, *Avr3a*, and *Avr6*) in order to diagnose with precision the pathotypes of *P. sojae* isolates. For this purpose, we analysed the genomic regions of these *Avr* genes in 31 recently sequenced isolates with different virulence profiles and identified discriminant mutations between avirulence and virulence alleles. Specific primers were designed to generate amplicons of a distinct size, and polymerase chain reaction conditions were optimized in a final assay of two parallel runs. When tested on the 31 isolates of known virulence, the assay accurately revealed all avirulence alleles. The test was further assessed and compared to a phenotyping assay on 25 isolates of unknown virulence. The two assays matched in 97% (170/175) of the interactions studied. Interestingly, the sole cases of discrepancy were obtained with *Avr3a*, which suggests a possible imperfect interaction with *Rps3a*. This molecular assay offers a powerful and reliable tool to exploit and study with greater precision soybean resistance against *P. sojae*.

## Graphical abstract

This study describes a molecular assay capable of defining the pathotypes of *P. sojae* and offers a powerful and reliable tool to exploit and study with greater precision soybean resistance against *P. sojae*.

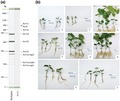

## 1 INTRODUCTION

Soybean (*Glycine max*) production has steadily increased all over the world and particularly in Canada over the last decade, and it is now the third most important crop in the country, covering more than 3 million hectares (Statistics Canada, 2018). This rapid expansion has led to increased disease pressure from a variety of pathogens. Among them, *Phytophthora sojae*, the causal agent of phytophthora root rot (PRR), is arguably the most economically important soybean pathogen in Canada (Xue *et al*., 2015). *P. sojae* is an oomycete belonging to the kingdom of Stramenopiles. It is a filamentous eukaryotic organism related to brown algae, but it does not perform photosynthesis. Like other oomycetes, morphologically it strongly resembles fungi (Tyler, 2007), but its hyphae, unlike those of fungi, are non‐partitioned and consist of cellulose rather than chitin (Bartnicki‐Garcia, 1968).

The pathogen can infect soybean at all growth stages, from seed to adult plants (Tyler, 2007), and can cause damage at any time in the season. Symptoms include seed rot, damping‐off, browning, and death of the roots and stem. Sometimes the foliage can turn brown and wither. Conditions favourable to the development of this disease are poor drainage, frequent in clay soils, which can easily be flooded for a longer period. Under favourable conditions, the oospores, the survival structure of *P. sojae*, will germinate into sporangia containing zoospores that can move easily in water with their two flagella. These motile spores are attracted to the root exudates of soybean by chemotaxis. Once they reach the roots, the zoospores are able to penetrate and invade the root cells with a structure called a haustorium. Following sexual reproduction in the root cortex, new oospores are formed and can survive in the dead plant or tissues for many years even under adverse conditions (Tyler, 2007).

Currently, the most effective method to curb the devastating impact of *P. sojae* is genetic resistance in the host. Complete (or vertical) resistance against *P. sojae* is conferred by single genes named *Rps* (resistance to *Phytophthora sojae*). These resistance genes act by recognizing the products of avirulence (*Avr*) genes of the pathogen in a traditional gene‐for‐gene relationship. The *Avr* genes of *P. sojae* encode effector proteins, most with RxLR (Arg‐X‐Leu‐Arg) motifs, and these effectors are recognized by proteins having nucleotide‐binding site and leucine‐rich repeat (NLR receptors) motifs, encoded by the *Rps* genes (Tyler and Gijzen, 2014).

The presence of *Rps* genes in soybean cultivars has resulted in an increased selection pressure, which in turn has led to the development of new pathotypes, of which 200 combinations have been reported in populations of *P. sojae* (Kaitany *et al*., 2001; Dorrance *et al*., 2004; Tyler and Gijzen, 2014). These new pathotypes reflect the occurrence of mutations in *P. sojae Avr* genes, whereby the latter are no longer recognized by *Rps* genes, opening the way to new infections (Shan *et al*., 2004; Rasoolizadeh *et al*., 2018). Typically, an *Rps* gene can be overcome and cease to be effective when the pathogen loses the ability to produce the corresponding Avr protein, following deletion or altered expression of an *Avr* gene, or alters its structure through point mutations or indels in the gene, making it unrecognizable by the product of the *Rps* gene (Tyler and Gijzen, 2014). Currently, there are approximately 25 known *Rps* genes in soybean, of which six have been introgressed internationally into commercial soybean varieties (*Rps1a*, *Rps1b*, *Rps1c*, *Rps1k*, *Rps3a*, and *Rps6*), concordant with *Avr* genes in *P. sojae* (Sugimoto *et al*., 2012; Abeysekara *et al*., 2016).

To properly exploit *Rps* genes against *P. sojae*, deployment of soybean cultivars with an appropriate combination of *Rps* genes, to match *Avr* genes present in field isolates, is essential. It is thus imperative to identify the virulence profile or pathotypes of these isolates present in a given environment by precisely detecting the presence or absence of functional *Avr* genes. For this purpose, phenotyping assays with soybean differentials carrying a single *Rps* gene have typically been exploited. The hypocotyl assay has been historically the most commonly used because of its simple protocol (Kaufmann and Gerdemann, 1958; Dorrance *et al*., 2008). However, problems with false positives, intermediate responses, and stability of *Avr* genes/expression over time have hampered its reliability. More recently, Lebreton *et al*. (2018) proposed a new assay to phenotype the isolates of *P. sojae* that relied on zoospore inoculation in hydroponic solutions to more closely reproduce the natural course of infection. While this bioassay appears to eliminate some of the shortcomings of the hypocotyl assay, it remains labour‐intensive and requires several weeks to complete.

In a recent effort to associate phenotypes with genotypes, Arsenault‐Labrecque *et al*. (2018) sequenced 31 *P. sojae* isolates collected from Canadian fields and analysed the sequences and variants of the seven most common *Avr* genes: *1a*, *1b*, *1c*, *1d*, *1k*, *3a*, and *6*. They were able to define specific haplotypes for each *Avr* gene and further show, through the use of the hydroponic assay, that each haplotype corresponded to a distinct virulence profile. This suggests that genomic signatures can be used as accurate predictors of phenotypes and could potentially be exploited as diagnostic tools to identify pathotypes in *P. sojae* isolates. On the basis of this hypothesis, the objectives of the present study were (a) to identify unique sequences based on the haplotypes identified by Arsenault‐Labrecque *et al*. (2018) that are specific and discriminant to detect the presence of the allele(s) conferring avirulence for each of the seven *Avr* genes, (b) to design primers from these sequences and to develop a molecular approach combining all the specific pairs of primers needed to amplify the seven avirulence alleles, and (c) to test the efficiency of the molecular tool by comparing the results with those obtained with a phenotyping assay. Here we describe a novel and unique diagnostic tool that defines with unprecedented precision the virulence profiles of *P. sojae* isolates. This tool can find direct applications in the study of functionality of *Avr* and *Rps* genes and in the development and selection of germplasm resistant to *P. sojae.*

## 2 RESULTS

### 2.1 Gene‐specific PCR‐based markers for seven *Avr* genes in *P. sojae*

**Figure 1:**
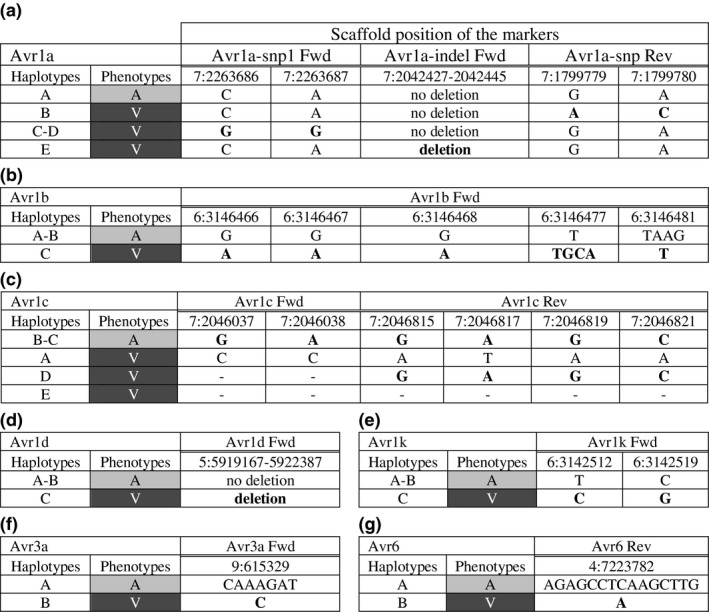
Discriminant haplotypes associated with distinct phenotypes in seven avirulence genes of *Phytophthora sojae* used to build discriminant primers: (a) *Avr1a*, (b) *Avr1b*, (c) *Avr1c*, (d) *Avr1d*, (e) *Avr1k*, (f) *Avr3a*, and (g) *Avr6*. A = avirulent and V = virulent. Haplotype letters refer to those previously identified by Arsenault‐Labrecque *et al*. (2018). Scaffold positions refer to the position of the markers used as per the *P. sojae* reference genome v. 3.0

For all seven *Avr* genes under study, all primers were designed to amplify sequences associated with the avirulence allele of the genes (Figure 1). In some cases, markers defined by Arsenault‐Labrecque *et al*. (2018) were used directly, and in cases where markers were not discriminant enough for polymerase chain reaction (PCR) (e.g., one single nucleotide polymorphism [SNP]), additional markers associated with the same alleles were used for the design of the primers. In other cases where the discriminant variants were located outside of the coding region, the primers were developed based on the specific haplotype linked to the avirulence allele. The positions of all the amplified regions are shown in Table 1.

**Table 1:** Primer sequences, with their genomic positions, amplicon size and concentrations, used for the detection of seven *Avr* genes in the *Phytophthora sojae* genome

Primer names	Amplicon position^a^	Product size	Forward sequence^b^	Reverse sequence	Concentrations F and R primers (µM)
Avr1a‐indel	Scaffold_7:2,042,431–2,042,664	234	GAAAGTGGACGGATATTTTCAAC	CAAGGACGGACTGGTACAGA	0.100
Avr1a‐snp1	Scaffold_7: 2,263,667–2,263,879	213	CTTAGTGTGCACCAACAGCCA	ACCACACTTCACGGAGCATT	0.100
Avr1a‐snp2	Scaffold_7: 1,799,519–1,799,796	278	GCTTTTCATCCAACGCTCAT	AATGATTGGCGGCAGATC	0.150
Avr1b	Scaffold_6:3,146,464–3,146,866	403	AAGGGGTACAGCCTGGATAAG	CTTGCGCTGTGAAGTGTCAT	0.150
Avr1c	Scaffold_7:2,046,020–2,046,821	802	CGGCAGAAGTTCTGGAAGA	GCCTTCCTTTGTCAGATTCG	0.250
Avr1d	Scaffold_5:5,919,385–5,919,881	497	CACGAGCAATGTCCTGTACG	CGAGCGTCCGATTTATAACTGG	0.075
Avr1k	Scaffold_6: 3,142,499–3,142,801	303	CTGTTCAGAAACTTCCGGTGC	CATGAAAAAGTCGGGGTTTG	0.150
Avr3a	Scaffold_9: 615,324–615,930	607	CTAGGCAAAGATGTCACCTG	ATCATGGCAAGCACCAATCT	0.100
Avr6	Scaffold_4: 7,223,071–7,223,796	726	GTCGTGCTGCATACTCTTGG	CAAGCTTGAGGCTCTGTGCT	0.100

a Amplicon position is based on *P. sojae* reference genome v. 3.0.

b Underlined nucleotides indicate discriminant nucleotide positions used to design primers.

For *Avr1a*, multiple variants were found on alleles conferring virulence on soybean lines carrying the *Rps1a* gene (Figure 1a). One such variant was an 18‐bp deletion conferring virulence to all isolates carrying it. For those virulent isolates lacking the deletion, they were distinguished from the avirulent ones on the basis of two adjacent SNPs found in two separate regions (Avr1a‐snp1 and Avr1a‐snp2).

In the case of *Avr1b*, a combination of SNPs and indels, located within 15 bp of each other, was found to discriminate the avirulence allele (Figure 1b). A forward primer was thus designed in that region to encompass all five variants. Sequence alignments with isolates P6497, P7064, P7074, and P7076 showed that the primers would accurately predict the phenotypes for the latter three isolates (Figure S1), isolate P6497 being a rare outlier as reported by Arsenault‐Labrecque *et al*. (2018).

The *Avr1c* avirulence allele could be discriminated from the virulence form on the basis of two SNPs situated at the 3′ end of the forward primer and four SNPs positioned at the 5′ end of the reverse primer (Figure 1c). This design allowed the avirulent haplotypes to be targeted specifically against several other haplotypes linked to virulence. When the reverse primer was aligned against the new haplotypes recently reported in Chinese isolates (Yang *et al*., 2019), our results demonstrated that it was specific to all avirulence alleles. In addition, it was specific to virulence allele *Avr1c‐4* but it is unknown if it would be amplified because we could not compare in silico alignment of the forward primer in absence of the sequences located outside the genic region.

In the case of *P. sojae* isolates carrying *Avr1d*, they were easily distinguished from those with pathotype 1d on the basis of a complete deletion of the gene (Figure 1d). Primers were thus simply designed to amplify a region within the gene.

For *Avr1k*, two SNPs were selected within 7 bp of each other that discriminated the avirulence allele from the virulence one to design the primers (Figure 1e).

Based on two distinct haplotypes, the avirulence allele of *Avr3a* presented an extra sequence of six nucleotides (Figure 1f). This area was therefore selected to design discriminant primers.

Finally, a 15‐bp deletion upstream of *Avr6* was consistently observed in all virulent isolates (Figure 1g). As it was consistently associated with a phenotype of virulence, it was used for primer design.

### 2.2 Analysis of genetic diversity

Based on availability of sequences covering the zones defined by our primers, *Avr1k* and *Avr6* were compared between isolates used in this study with those of isolates from China and the United States. This phylogenetic analysis revealed that they encompassed the genetic diversity currently reported for those genes (Figure S2). Interestingly, all outside isolates used for comparison matched the expected virulent phenotype except for a single isolate, which was reported as virulent (with the hypocotyl assay) despite carrying the avirulence allele.

### 2.3 Uniplex PCR amplification and specificity

**Figure 2:**
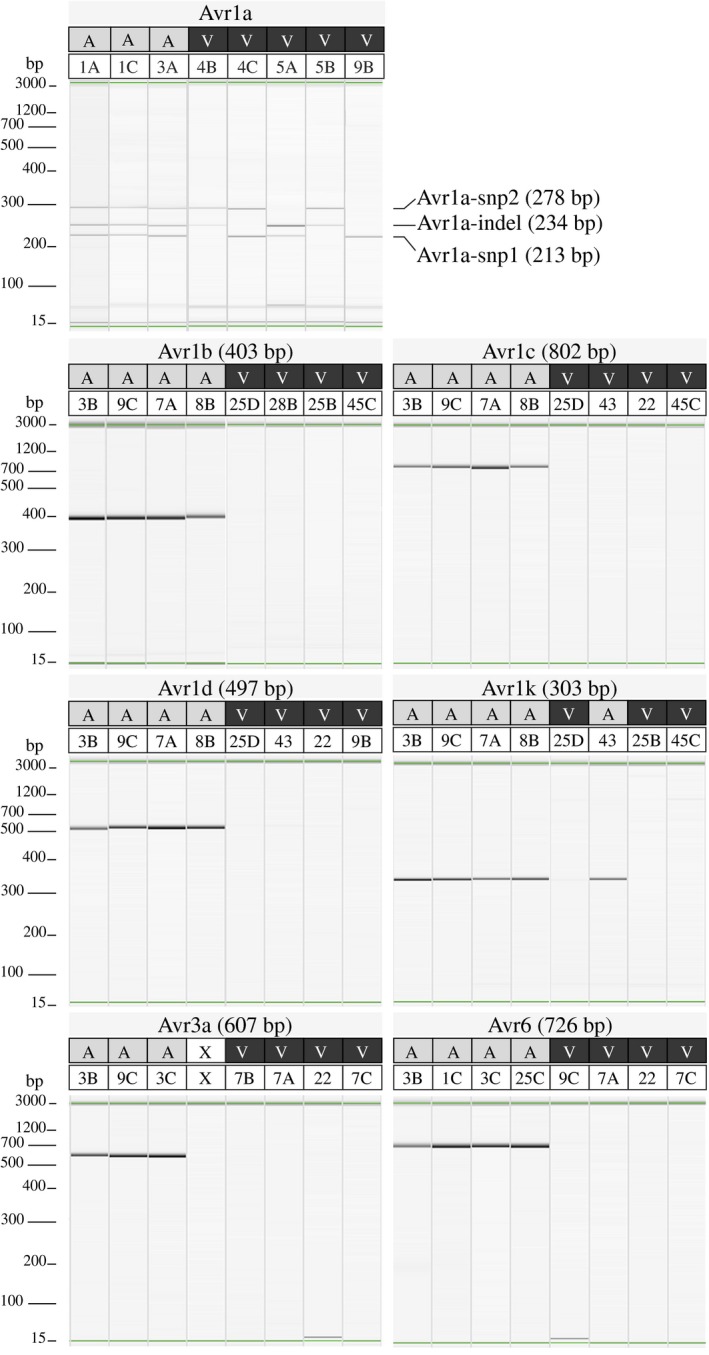
Gel images of uniplex polymerase chain reaction amplifications of discriminant regions associated with avirulence alleles for seven *Avr* genes in *Phytophthora sojae*. For each *Avr* gene, the expected phenotype (A = avirulent and V = virulent) against the corresponding *Rps* gene for each of the eight isolates tested is indicated at the top of the gel. The expected size of the amplicon is noted in parentheses next to each *Avr* gene, except for *Avr1a*. For *Avr1a*, three distinct regions, Avr1a‐indel, Avr1a‐snp1, and Avr1a‐snp2, must amplify to detect a phenotype of avirulence

The results showed that successful amplification of the functional version of the *Avr* genes matched perfectly the expected phenotype for each of the seven *Avr* genes, thus confirming the specificity of the primers for the targeted region (Figure 2). For six of the seven genes, a single set of primers was sufficient to discriminate the haplotypes leading to a virulent or avirulent reaction. In the case of *Avr1a*, four different haplotypes were exploited so three different pairs of primers had to be designed and included in the molecular assay to cover the spectrum of possible haplotypes: Avr1a‐indel, Avr1a‐snp1, and Avr1a‐snp2 (Figure 2). As explained above, only the simultaneous presence of all three amplicons indicated avirulence.

### 2.4 Multiplex PCR and specificity

**Figure 3:**
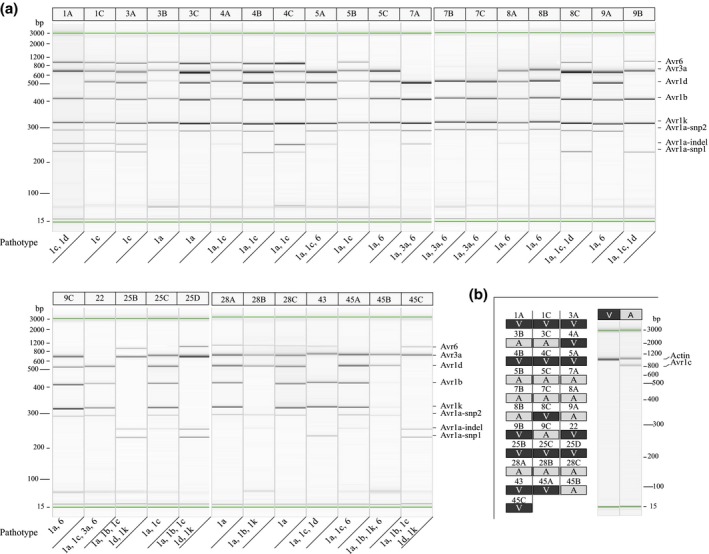
Gel images of multiplex polymerase chain reaction (PCR) amplifications of discriminant regions associated with avirulence alleles for seven *Avr* genes in *Phytophthora sojae*. (a) Results obtained with 31 isolates with a known pathotype, as indicated at the bottom of the gel, for *Avr1a*, *1b*, *1d*, *1k*, *3a*, and *6*. The expected size of the amplicon for each *Avr* gene is indicated on the right. (b) Complementary gel of PCR amplification of discriminant region associated with the avirulence allele for *Avr1c* (right) along with results obtained for the 31 isolates (A = avirulent and V = virulent) where A or V indicates presence or absence of the amplicon, respectively. For each isolate, the pathotype should correspond to the absence of an amplicon for each corresponding gene

Following optimization of the PCR conditions, the molecular assay was carried out in two parallel runs: one multiplex assay for the detection of *Avr1a, Avr1b, Avr1d, Avr1k, Avr3a*, and *Avr6*, and one singleplex assay for *Avr1c*. The presence of a band, or three in case of *Avr1a*, of a specific size as described in Table 1 indicates that the tested isolate carries the avirulence allele associated with the amplicon of the *Avr* gene of that size. Conversely, the absence of an amplicon for a given gene indicates that the isolate carries the corresponding virulence allele. For instance, Figure 3 presents results from the multiplex PCR assay on the 31 known isolates with their corresponding pathotype based on a phenotypic assay (Arsenault‐Labrecque *et al*., 2018). Results show that the pathotype, as expressed by the absence of an amplicon for a given gene, is accurately predicted by the molecular assay. As an illustration, isolate 1A shows amplicons for *Avr1a, 1b, 1k, 3a* and *6* (Figure 3a) and none for *1d* and *1c* (Figure 3b), which translates into pathotype 1c and 1d.

### 2.5 Genotyping and phenotyping of isolates with unknown pathotype

**Figure 4:**
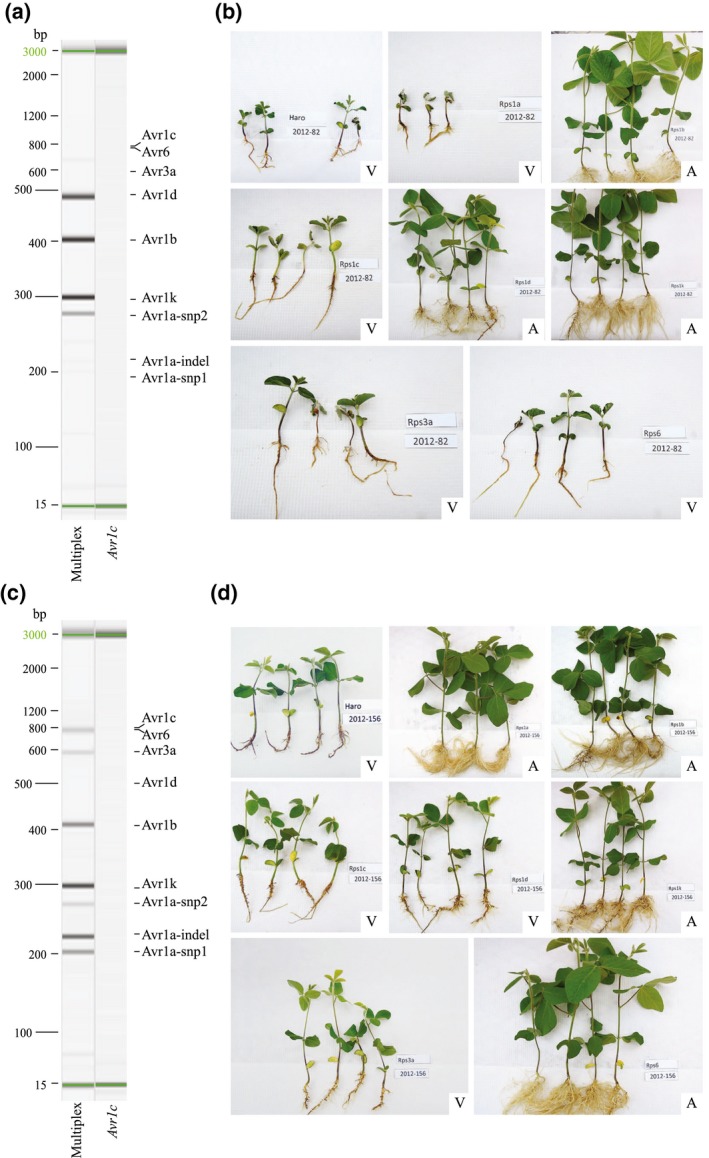
Comparison of molecular and phenotyping assays to determine the pathotypes of *Phytophthora sojae* isolates. (a) Gel image of multiplex polymerase chain reaction (PCR) amplifications of discriminant regions associated with avirulence alleles for seven *Avr* genes in *P. sojae* isolate 2012–82. Presence of amplicons for *Avr1b*, *1d*, and *1k* predicts a pathotype 1a, 1c, 3a, and 6. (b) Phenotyping results for isolate 2012–82 indicates a compatible interaction with Harosoy (*rps*), *Rps1a*, *Rps1c*, *Rps3a*, and *Rps6* and an incompatible interaction with *Rps1b*, *Rps1d*, and *Rps1k*, thereby assessing a pathotype 1a, 1c, 3a, and 6, similar to the molecular assay. A = avirulent and V = virulent (see Figure S3). (c) Gel image of multiplex PCR amplifications of discriminant regions associated with avirulence alleles for seven *Avr* genes in *P. sojae* isolate 2012–156. Presence of amplicons for *Avr1b*, *1k*, *3a*, and *6* and all three amplicons for *Avr1a* predicts a pathotype 1c and 1d. (d) Phenotyping results for isolate 2012–156 indicates a compatible interaction with Harosoy (*rps*), *Rps1c*, *Rps1d*, and *Rps3a* and an incompatible interaction with *Rps1a*, *Rps1b*, *Rps1k*, and *Rps6* thereby assessing a pathotype 1c, 1d, and 3a, with 3a being the only interaction at odds with the molecular assay. A = avirulent and V = virulent (see Figure S3)

After validation of the multiplex PCR assay with the 31 known isolates, 25 uncharacterized isolates were randomly selected to confirm the effectiveness of the assay. Representative results obtained following the molecular and hydroponic assays are presented for two isolates (Figure 4). The seedlings were scored and values compared to those of Haro (susceptible control) using Dunnett's test to determine if they were susceptible or resistant (Figure S3). As seen in Figure 4a, the presence of amplicons for *Avr1b, Avr1d*, and *Avr1k* on the gel is indicative that isolate 2012–82 should have pathotype 1a, 1c, 3a, 6. When compared with the bioassay (Figure 4b), the phenotypes obtained clearly corroborated the molecular assay where a compatible interaction was observed between the isolate and differentials *Rps1a*, *1c*, *3a*, and *6*. In the other example with isolate 2012–156 (Figure 4c), the molecular assay showed amplification for *Avr1a* (Avr1a‐snp1, Avr1a‐indel, Avr1a‐snp2), *1b, 1k*, *3a*, and *6*, which leads to a diagnostic of pathotype 1c, 1d for that isolate. Interestingly, the phenotypic assay shown in Figure 4d confirmed the compatible interaction with differentials *Rps1c* and *1d* but also suggested one with *Rps3a*, despite the molecular assay clearly showing an amplicon for *Avr3a*. As a matter of fact, when results were combined for all 25 isolates and seven *Avr* genes (175 interactions), there was only a single and similar discrepancy when the molecular assay and the phenotypes did not match perfectly (Table 2). Indeed, in five cases a compatible interaction was observed with *Rps3a* in the hydroponic assay despite the presence of an amplicon for the avirulence allele of *Avr3a*. All other interactions generated a perfect match between the molecular and the phenotyping assay for a prediction accuracy of 97% (170/175).

**Table 2:** Comparative results of predicted pathotypes between the molecular assay and the hydroponic assay for 25 isolates of *Phytophthora sojae*

Isolate	Predicted pathotype (molecular assay)	Observed pathotype^a^ (hydroponic assay)
2010–29	1a, 1c	1a, 1c, 3a
2010–32	1a, 1c	1a, 1c
2010–42	1a, 1c	1a, 1c
2010–44	1a, 1c	1a, 1c, 3a
2011–35	1a, 1c, 3a	1a, 1c, 3a
2011–40	1a, 1b, 1c, 1k	1a, 1b, 1c, 1k
2012–01	1a, 1c, 3a, 6	1a, 1c, 3a, 6
2012–120	1a, 1c, 6	1a, 1c, 6
2012–127	1a, 1c	1a, 1c
2012–136	1a, 1c, 6	1a, 1c, 6
2012–156	1c, 1d	1c, 1d, 3a
2012–40	1a, 1c, 6	1a, 1c, 6
2012–57	1a, 1c, 1d	1a, 1c, 1d
2012–76	1a, 1c	1a, 1c, 3a
2012–82	1a, 1c, 3a, 6	1a, 1c, 3a, 6
2016–20	1a, 1b, 1c, 1d, 1k	1a, 1b, 1c, 1k, 1d
2018–01	1a, 1c, 1d, 3a	1a, 1c, 1d, 3a
2018–02	1a, 1b, 1c, 1d, 1k	1a, 1b, 1c, 1d, 1k
2018–03	1a, 1b, 1c, 1d, 1k	1a, 1b, 1c, 1d, 1k, 3a
2018–04	1a,1b, 1c, 1d, 1k, 3a	1a, 1b, 1c, 1d, 1k, 3a
2018–05	1a, 1c, 1d, 3a	1a, 1c, 1d, 3a
2018–06	1a, 1b, 1c, 1d, 1k	1a, 1b, 1c, 1d, 1k
2018–07	1a, 1c, 1d, 3a	1a, 1c, 1d, 3a
2018–08	1a, 1b, 1c, 1d, 1k	1a, 1b, 1c, 1d, 1k
2018–09	1a, 1c, 1d, 3a	1a, 1c, 1d, 3a

a Underlined pathotype indicates discrepancy with the molecular assay.

## 3 DISCUSSION

In recent years, molecular techniques have revolutionized and greatly facilitated the diagnostic of plant pathogens (Kostov *et al*., 2016; Michalecka *et al*., 2016; Reich *et al*., 2016; Silva *et al*., 2017; Burbank and Ortega, 2018). Among other applications, they have allowed the precise identification of the causal agents of diseases caused by a complex of fungi (Conti *et al*., 2019), the fulfillment of Koch's postulates with closely related species such as *Fusarium* spp. (Moine *et al*., 2014), and the diagnosis of the presence and identity of a pathogen from levels that were hitherto undetectable (Rollins *et al*., 2016; Haudenshield *et al*., 2017). In most cases, those PCR‐based assays have exploited conserved regions such as the internal transcribed spacers (ITS) (Bruns and Shefferson, 2004) or the translation elongation factor 1α (TEF1‐α) to develop diagnostic tools able to identify a given pathogen (Raja *et al*., 2017; Marin‐Felix *et al*., 2019). By contrast, to our knowledge, this study presents the first molecular assay aimed at identifying *Avr* genes for the purpose of diagnosing the virulence profile of a plant pathogen. To this end, this assay necessitated access to the full genome sequences of many isolates of *P. sojae* in order to capture the spectrum of diversity within each *Avr* gene under study. Indeed, genetic control of *P. sojae* through introgression of *Rps* genes in soybean presupposes a thorough understanding and knowledge of the pathotype diversity of *P. sojae* isolates in the specific area where the *Rps* genes are deployed. Up to this point, the only way to determine the pathotype of a given isolate was through cumbersome and long phenotyping procedures, each with its own shortcomings (Dorrance *et al*., 2008; Lebreton *et al*., 2018). Through this unique molecular assay, based on discriminant haplotypes for seven *Avr* genes of *P. sojae*, it should now be possible to obtain a rapid and accurate identification of the virulence profile of isolates in order to precisely select soybean material carrying the appropriate *Rps* genes.

Since the intentional deployment of the first *Rps* gene in soybean, *Rps1a*, *P. sojae* has demonstrated a very strong resilience and ability to adapt to selection pressure through rapid mutations of *Avr* genes (Keeling, 1984; Layton *et al*., 1986; Drenth *et al*., 1996; Kaitany *et al*., 2001). As a result, the pathogen has evolved a staggering pathotype diversity (Dorrance *et al*., 2003; Sugimoto *et al*., 2012; Dorrance *et al*., 2016; Dorrance, 2018) that threatens current efforts to control its spread through genetic approaches. For instance, in a survey of *P. sojae* isolate diversity in Canada, Xue *et al*. (2015) reported an important shift in virulence over time, whereas most isolates now overcome *Rps1k*, the most recently introduced *Rps* gene, while this pathotype was completely absent in Canadian fields 20 years ago. This ability to alter *Avr* genes appears to be based on mutations that range from complete deletion of the gene or copy number variation, to presence of indels or single point mutations within or in close proximity to the gene (Qutob *et al*., 2009; Tyler and Gijzen, 2014). Recently, Arsenault‐Labrecque *et al*. (2018) provided an exhaustive description and comparison of haplotype diversity for seven *Avr* genes (*1a*, *1b*, *1c*, *1d*, *1k*, *3a*, and* 6*) of *P. sojae* through whole‐genome sequencing of 31 isolates with different pathotypes. While all isolates for the study originated from Canada, their results showed that they had captured all the haplotype diversity previously reported (but for one rare allele in *Avr1b* [Shan *et al*., 2004]) and even expanded it for a few *Avr*s. In this work, we further compared the allelic diversity of two avirulence genes (*Avr1k* and *Avr6*) among isolates from China, the United States, and Canada and found that all alleles were well aligned with the predicted virulence, except for a single isolate phenotyped with the hypocotyl assay, indicating that the accuracy of the bioassay expands to most known *P. sojae* isolates, regardless of their origin (Figure S2). In the case of *Avr1b*, our study showed that out of the four strains P6497, P7064, P7074, and P7076, only P6497 carried a haplotype of avirulence and showed a phenotype of virulence (Figure S1). However, it is important to underline that P6497 represents a rare case of virulence against *Rps1b* and avirulence against *Rps1k*, and that its pathotype is uncommon in nature (Xue *et al*., 2015). For these reasons, the sequences obtained by Arsenault‐Labrecque *et al*. (2018) offered a precise blueprint of the currently known sequence variation and conservation in each gene, results that we exploited to identify discriminant regions associated with different phenotypes.

In the process of trying to develop a molecular assay based on the discriminant haplotypes, several challenges were encountered at different stages of the study. First, it is known that the acquisition of virulence for a pathogen is often due to a partial or complete deletion of the *Avr* gene (Jones and Dangl, 2006; Guttman *et al*., 2014; Petit‐Houdenot and Fudal, 2017). In our study, this phenomenon was only systematically observed in the case of *Avr1d*, which meant that we had to conduct an exhaustive analysis of the upstream and downstream regions of the other *Avr* genes to find SNPs/indels that would segregate haplotypes associated with avirulence from those associated with virulence. In certain instances, as for *Avr1a*, *1c*, and *6*, the discriminating regions were located outside of the coding region of the gene.

When the molecular assay was applied to the 31 isolates that had been previously phenotyped (Arsenault‐Labrecque *et al*., 2018), we were able to show a near‐perfect adequation between the two approaches. This confirms that the molecular assay constitutes a valid substitute to the long phenotyping assays and offers a much more practical approach to determine pathotypes of *P. sojae* isolates. It can thus find applications in delineating with precision the deployment of soybean lines carrying the proper *Rps* genes to overcome the pathotypes present in a given environment. Furthermore, as new resistance genes are discovered and introgressed into soybean, the test can be adapted to include new *Avr* genes and follow the evolution of new pathotypes over time. Finally, it can incite the development of similar diagnostic tools for other gene‐for‐gene dependent plant–pathogen interactions.

On testing the accuracy of the molecular assay with 25 isolates of unknown virulence, some interesting biological insights were highlighted with respect to some *Avr* genes. As a first observation, it was certainly validating to record a 97% (170/175) level of concordance between the molecular and the phenotyping assays. Of additional significance is the fact that the only five cases of discrepancy out of 175 interactions tested were related to *Avr3a*, which gave a false predicted phenotype, consistently erring on the side of a virulent response where avirulence was expected based on the molecular assay. This was in clear contrast with the study of Arsenault‐Labrecque *et al*. (2018) where *Avr3a* was the only gene where the two haplotypes aligned perfectly with a phenotype of virulence or avirulence as determined by the hypocotyl assay. As a result, *P. sojae* isolates were never tested, until this study, with the hydroponic assay against a differential carrying *Rps3a*. Reasons as to why *Avr3a* appears so unpredictable in our study can fall into two categories. The first one is related to gene regulation, which would explain that presence of the *Avr* gene with repressed expression can lead to a virulent response (Shan *et al*., 2004). It is well known that *Avr* genes are under strong selection pressure and their expression can potentially be altered by a number of events (Wang *et al*., 2011). Incidentally, Arsenault‐Labrecque *et al*. (2018) did observe cases where lower expression of *Avr1c* led to a phenotype of virulence. In addition, Qutob, *et al*. (2013) showed that the level of expression of *Avr3a* could be altered by the presence of small RNAs that would lead to silencing of the gene. Gijzen *et al*. (2014), suggested that epigenetic factors could alter *Avr* gene expression. However, the literature is still rather scant with respect to expression of *Avr3a*, and this phenomenon, if common, needs to be further studied. Alternatively, another explanation could lie in the efficiency of *Rps3a*. *Rps3a* is the least deployed of *Rps* genes accounting for about only 0.3% in commercially released varieties (Slaminko *et al*., 2010). Field observations have also reported the somewhat random nature of its efficiency (Dorrance *et al*., 2003). The different phenotypes obtained between the hydroponic assay and the hypocotyl assay seem to indicate that *Rps3a*, if effective at the collar, does not confer good root resistance, which would also explain its irregular field performance. These results certainly open the way to investigate more precisely the nature of *Rps3a* and its interaction with *Avr3a*.

In a recent report, Yang *et al*. (2019) described new haplotypes of the *Avr1c* gene from isolates recovered in Chinese fields. It was therefore interesting to determine if the primers designed in this study would discriminate those new haplotypes. While in the absence of the sequences for the forward primer it is impossible to make a definitive conclusion, the reverse primer was nonetheless specific to all avirulence alleles. Only virulence allele *Avr1c‐4* also showed specificity so it will be worthwhile to determine if the forward primer would discriminate it as is the case with the virulent haplotype D (Arsenault‐Labrecque *et al*., 2018), which is also specific to the reverse primer but not the forward one. In the event that the existing forward primer would not discriminate *Avr1c‐4*, another primer could easily be designed that captured both haplotype D and *Avr1c‐4*.

In summary, this work describes a comprehensive molecular assay capable of defining the pathotypes of *P. sojae*, based on seven *Avr* genes, with unprecedented ease and precision. Its other advantages can be found in eliminating the shortcomings of the different phenotyping procedures while reducing time and resources involved in the process. The test could be further exploited to study and uncover the molecular phenomena underlying the disparity between absence/presence of avirulence alleles and expected phenotypes. It can also have practical applications for breeders and growers in management of the disease with a tailored deployment of use of *Rps* genes based on a precise and rapid determination of pathotypes present in a given area.

## 4 EXPERIMENTAL PROCEDURES

### 4.1 *Phytophthora sojae* isolates and DNA extraction

All *P. sojae* isolates used in this study, including 31 previously sequenced by Arsenault‐Labrecque *et al*. (2018), were sampled across Quebec and Ontario (Canada) and their virulence profile was determined by the hypocotyl assay as reported by Xue *et al*. (2015). To determine if the avirulence genes of the isolates used in this study captured the genetic diversity of other isolates elsewhere in the world, we compared the sequences of two *Avr* genes by alignment and made phylogenetic trees using CLC Genomics Workbench (Qiagen). These two genes were selected because they offered the largest sequence diversity available in the literature and public database (Dou *et al*., 2010; Song *et al*., 2013; Arsenault‐Labrecque *et al*., 2018). In addition, Yang *et al*. (2019) recently reported new haplotypes of the *Avr1c* gene from isolates recovered in Chinese fields. To verify if the primers created for *Avr1c* were discriminant for these new alleles, alignments were performed with CLC Genomics Workbench with the reverse primer only, because sequences for the forward primer, located outside the genic region, were not available.

The isolates were first subcultured on V8 agar medium (20% clarified V8) covered with wax paper to facilitate harvest of hyphae and spores. After 1 week, cultures were scraped off the paper with a scalpel and placed in 1.5‐mL tubes with screw caps (OMNI International Inc.). The tubes were then kept in the freezer at −80 °C for 2–3 hr and lyophilized overnight. The lyophilized samples were crushed with an Omni Bead Ruptor 24 (OMNI International). The DNA was then extracted from the crushed samples using the E.Z.N.A Plant DNA kit following the manufacturer's protocol for dried samples with slight modifications.

### 4.2 Sequence variations and allele‐specific primer design

For designing allele‐specific primers, the discriminant variations in the sequences of the different *Avr* genes of 31 isolates were studied and identified based on the *P.sojae* reference genome v. 3.0 and the genomic sequences available in the NCBI SRA repository, under the bioproject PRJNA434589 as reported by Arsenault‐Labrecque *et al*. (2018). In all cases, we sought to obtain amplicons associated with the avirulence allele(s) and of different sizes such that primers could be used in a multiplex assay and the amplicons easily resolved via gel electrophoresis. Discriminant variations most convenient for marker development were selected to design the primer pairs for the seven *Avr* genes under study (*Avr1a*, *1b*, *1c*, *1d*, *1k*, *3a*, and *6*). In cases where deletions were present, at least one primer was positioned in the deletion such that the avirulence allele (i.e., without the deletion) could be amplified. If only SNPs differentiated the virulence and avirulence alleles, primers were designed in such a way that these variant positions were located at the 3′ extremity to maximize the specificity of amplification. Regions with two or more SNPs were preferentially selected to increase the allelic specificity. The primers were then synthesized by Thermo‐Fisher Scientific. The details of the nine pairs of primers are presented in Table 1.

### 4.3 Validation of primer specificity

A simple PCR was first conducted with each of the primer pairs individually to ascertain their specificity. The PCR was performed in a T Professional Thermocycler (Biometra) and was carried out in a reaction volume of 20 µL. Each primer was diluted at a concentration of 0.25 µM. The One Taq NEB (New England Biolabs) was used at 0.025 U/µL with 2 µL of DNA extracted from *P. sojae* at a concentration of 10 ng/µL, 5 × One Taq Standard reaction buffer (New England Biolabs), 0.2 mM dNTPs, and 2.5% DMSO (Sigma). The PCR conditions were as follows: an initial denaturation at 94 °C for 5 min followed by 30 cycles of denaturation at 94 °C for 30 s, annealing at 60 °C for 30 s, elongation at 68 °C for 1 min, and a final elongation at 68 °C for 5 min. DNA fragment analysis was performed using a QIAxcel Advanced System on a DNA high‐resolution cartridge, based on method OH500 with alignment markers of 15 and 3,000 bp according to the manufacturer's instructions (Qiagen). A PCR was performed on each of the 31 isolates of *P. sojae* with a known pathotype to validate that the presence of the expected amplicon was associated with an avirulent response.

In addition, because *Avr1b* has been reported as potentially affecting the virulence of other avirulence genes (Dou *et al*., 2008), we performed in silico alignments with CLC Genomics Workbench to validate if the primer for *Avr1b* would accurately predict the phenotypes for the well‐studied isolates P6497, P7064, P7074, and P7076 (Shan *et al*., 2004).

### 4.4 Optimization of the multiplex PCR

Following optimization of primer concentration, annealing temperature, and dNTP concentration, primers were mixed together in a single PCR to check their compatibility in a multiplex PCR. It was found that the primers amplifying the *Avr1c* gene were not compatible with the other primers because, when mixed together, primer dimers were formed. Attempts to design alternative sets of primers were unsuccessful, so it was decided that the primers for *Avr1c* would be used in a separate assay in parallel with the multiplex assay. The multiplex PCR therefore contains the following eight primer sets: Avr1a‐indel, Avr1a‐snp1, Avr1a‐snp2, Avr1b, Avr1d, Avr1k, Avr3a, and Avr6.

The optimal number of cycles for the reaction was 40. Furthermore, a temperature gradient revealed that the temperature obtaining the most intense and most distinct bands was 55 °C for the multiplex PCR and 60 °C for the uniplex PCR. The dNTP concentration chosen was 0.25 mM. The final PCR products were analysed with the QIAxcel Advanced System (Qiagen).

The PCRs were carried out in a reaction volume of 20 µL. Following several tests of dilution of the primers, each primer was diluted at the optimal concentration detailed in Table 1. The One Taq (New England Biolabs) was used at 0.025 U/µL with 2 µL of DNA at a concentration of 10 ng/µL, 5 × One Taq Standard reaction buffer (New England Biolabs), 0.25 mM dNTPs, and 2.5% DMSO (Sigma). The multiplex PCR conditions consisted of an initial denaturation at 94 °C for 5 min followed by 40 cycles of denaturation at 94 °C for 30 s, annealing at 55 °C for 30 s, elongation at 68 °C for 1 min, and a final elongation at 68 °C for 5 min. For the uniplex PCR (*Avr1c*), the conditions were the same, except for annealing at 60 °C.

### 4.5 Detection limits of the multiplex PCR

To determine the lowest concentration of DNA at which the multiplex and the uniplex PCR worked, dilutions from 0.01 pg to 20 ng were tested with the two PCR conditions described above. It was determined that the PCR multiplex could detect a DNA concentration of up to 0.2 ng, while the primers tested individually could detect a DNA concentration of 0.2 pg.

### 4.6 Specificity of the molecular tool and phenotyping

Once the multiplex PCR conditions were optimized, the 31 isolates with known haplotypes, previously sequenced by Arsenault‐Labrecque *et al*. (2018), were analysed to test the efficiency of the molecular assay. Subsequently, 25 isolates collected in the Canadian provinces of Quebec and Ontario between 2010 and 2018 were tested with the multiplex PCR and phenotyped using the hydroponic assay developed by Lebreton *et al*. (2018). For the hydroponic assay, zoospores were inoculated into a hydroponic system containing a nutrient solution diluted in water. Seven differential soybean lines were grown in the hydroponic system with a susceptible control (cv. Harosoy), and the virulence profile of the isolate tested was determined on the basis of which *Rps* genes resulted in immunity. Phenotypic responses (resistance or susceptibility) were recorded 14 days post‐inoculation. Plants were scored based on a 1 to 5 scale developed by Lebreton *et al*. (2018) where 1 = none to limited root symptoms and 5 = mortality and advanced root necrosis. A statistical comparison (Dunnett's test) was performed to determine the phenotype of each of the isolates. When values were statistically similar to the ones obtained with cv. Haro (*rps*), the isolate was considered virulent, and when values were statistically lower, the isolate was considered avirulent.

## Supporting information


**Figure S1** Sequence alignment of *Avr1b* for *Phytophthora sojae* isolates P6497, P7064, P7074, and P7076. Results show that the allele‐specific primer (green arrow) anneals only to P6497 and P7064, thereby predicting a phenotype of avirulence for P6497 and P7064, and a phenotype of virulence for P7074 and 7076

**Figure S2** Phylogenetic trees showing the diversity of *Phytophthora sojae* isolates in the United States, Canada, and China for A *Rps1k* and B *Rps6* genes. Virulence and avirulence alleles are circled and identified

**Figure S3** Susceptibility scoring of soybean plantlets inoculated with *Phytophthora sojae* isolates A 2012‐82 and B 2012‐156 complementing the phenotyping assay of Figure 4. Interactions are considered incompatible when values (indicated with *) are significantly different from the *rps *gene (susceptible control) according to Dunnett’s test (*p* < .01)

## Data Availability

The data that support the findings of this study are openly available in the NCBI SRA repository at http://www.ncbi.nlm.nih.gov/sra under the bioproject PRJNA434589.
