# Supplementary material for: Discriminant haplotypes of avirulence genes of Phytophthora sojae lead to a molecular assay to predict phenotypes
Source: Mol Plant Pathol. 2020 Jan 7;21(3):318–29. doi: 10.1111/mpp.12898 (PMC7036360; doi:10.1111/mpp.12898)
Supplement: Supplementary file 1 — Figure S1 Sequence alignment of Avr1b for Phytophthora sojae isolates P6497, P7064, P7074, and P7076. Results show that the allele‐specific primer (green arrow) anneals only to P6497 and P7064, thereby predicting a phenotype of avirulence for P6497 and P7064, and a phenotype of virulence for P7074 and 7076 [file MPP-21-318-s001.docx]

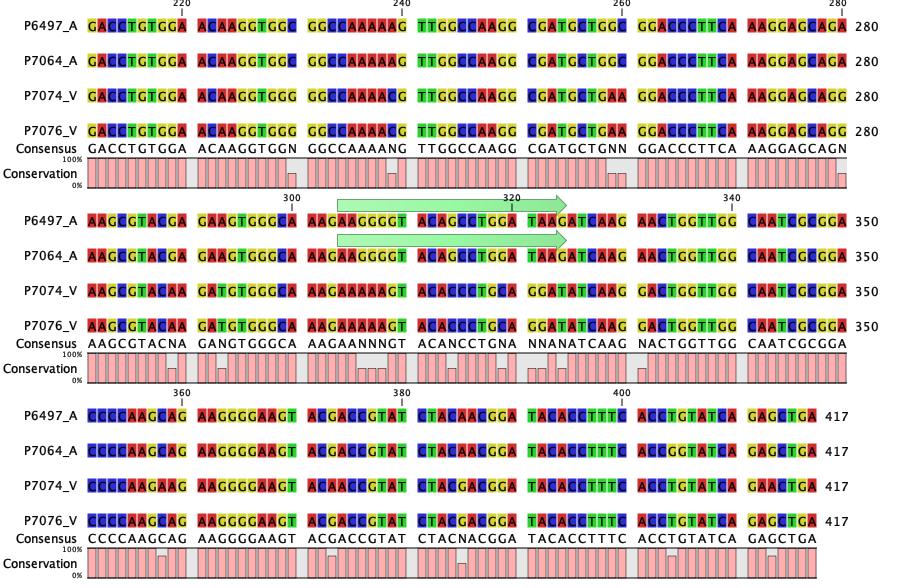


**Figure S1** Sequence alignment of *Avr1b* for *Phytophthora sojae* isolates P6497, P7064, P7074 and P7076. Results show that the allele-specific primer (green arrow) anneals only to P6497 and P7064, thereby predicting a phenotype of avirulence for P6497 and P7064 and a phenotype of virulence for P7074 and 7076.
