# Supplementary material for: Discriminant haplotypes of avirulence genes of Phytophthora sojae lead to a molecular assay to predict phenotypes
Source: Mol Plant Pathol. 2020 Jan 7;21(3):318–29. doi: 10.1111/mpp.12898 (PMC7036360; doi:10.1111/mpp.12898)
Supplement: Supplementary file 2 — Figure S2 Phylogenetic trees showing the diversity of Phytophthora sojae isolates in the United States, Canada, and China for A Rps1k and B Rps6 genes. Virulence and avirulence alleles are circled and identified [file MPP-21-318-s002.docx]

**Figure S2** Phylogenetic trees showing the diversity of *Phytophthora sojae* isolates in the United States, Canada, and China for **A** *Rps1k* and **B** *Rps6* genes. Virulent and avirulent alleles are circled and identified.
