# Supplementary material for: Discriminant haplotypes of avirulence genes of Phytophthora sojae lead to a molecular assay to predict phenotypes
Source: Mol Plant Pathol. 2020 Jan 7;21(3):318–29. doi: 10.1111/mpp.12898 (PMC7036360; doi:10.1111/mpp.12898)
Supplement: Supplementary file 3 — Figure S3 Susceptibility scoring of soybean plantlets inoculated with Phytophthora sojae isolates A 2012‐82 and B 2012‐156 complementing the phenotyping assay of Figure 4. Interactions are considered incompatible when values (indicated with *) are significantly different from the rps gene (susceptible control) according to Dunnett’s test (p < .01) [file MPP-21-318-s003.docx]

**A**

**B**


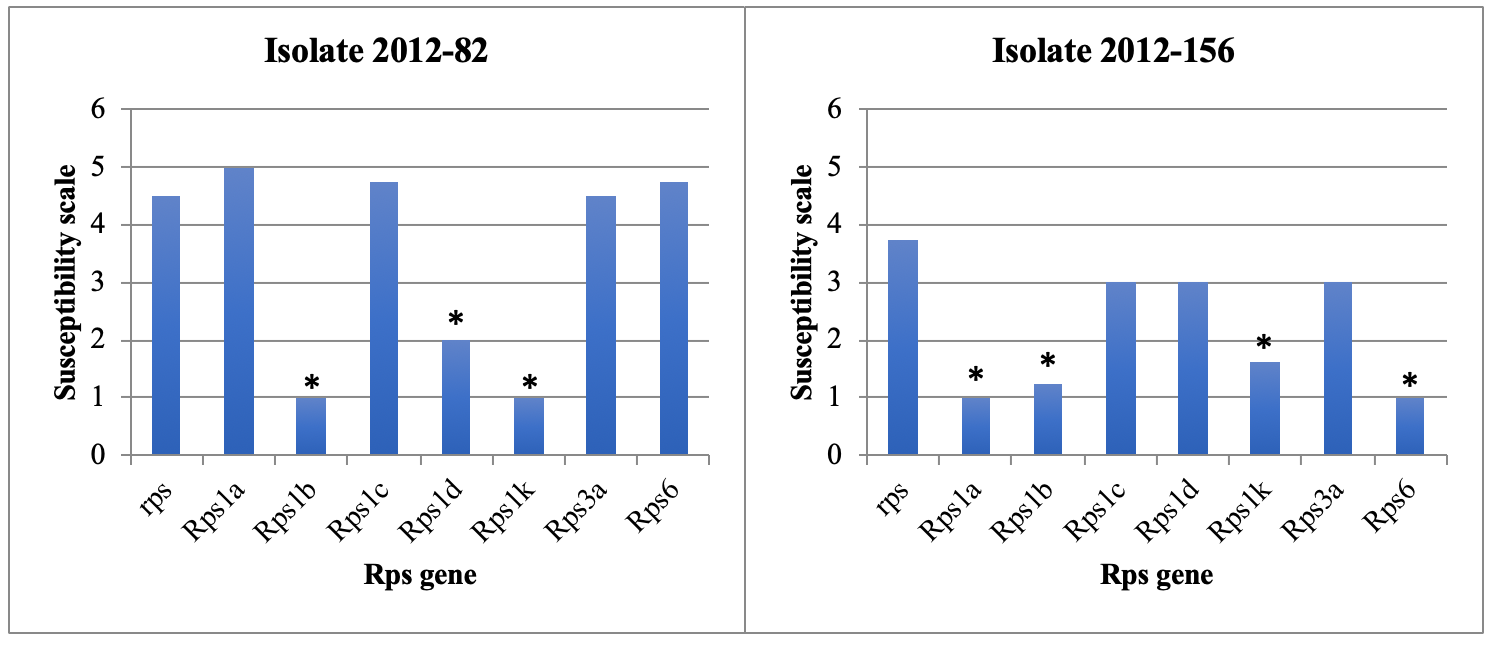


**Figure S3** Susceptibility scoring of soybean plantlets inoculated with *Phytophthora sojae* isolates **A** 2012-82 and **B** 2012-156 complementing the phenotyping assay of Figure 4. Interactions are considered incompatible when values (indicated with a *) are significatively different from the *rps* gene (susceptible control) according to Dunnett’s test (*P* < 0.01).
